# Super-Resolution 3D Imaging Reveals Disarray of Dyadic Calcium Ion Channels in Failing Hearts Expressing Low Thyroid Hormone Function

**DOI:** 10.3390/ijms27125601

**Published:** 2026-06-21

**Authors:** Atieh Ashkezari, Megha Schmalzle, Amanda Charest, Sanketh Kumar, Riddhi Modi, Nicholas Nasta, Andrea Bertolini, Alessandro Saba, Paolo Cifani, Youhua Zhang, A. Martin Gerdes, Randy F. Stout, Kaie Ojamaa

**Affiliations:** 1 Department of Biomedical Sciences, New York Institute of Technology College of Osteopathic Medicine, 600 Northern Blvd, Old Westbury, NY 11568, USA; adehgh02@nyit.edu (A.A.); mschma01@nyit.edu (M.S.); acharest@nyit.edu (A.C.); skumar38@nyit.edu (S.K.); rmodi07@nyit.edu (R.M.); nnasta@nyit.edu (N.N.); a.bertolini2@student.unisi.it (A.B.); yzhang49@nyit.edu (Y.Z.); martin.gerdes@nyit.edu (A.M.G.); 2 Department of Pathology, University of Pisa, Via Savi, 10, 56126 Pisa, Italy; alessandro.saba@unipi.it; 3 Mass Spectrometry Shared Resource, Cold Spring Harbor Laboratory, 1 Bungtown Rd., Cold Spring Harbor, NY 11724, USA; cifani@cshl.edu

**Keywords:** 3D STORM, ryanodine receptor-2, L-type calcium channel, junctophilin-2, thyroid hormone (T3)

## Abstract

Ventricular remodeling occurring in heart failure (HF) involves structural disarray of the sarcolemma T-tubule (TT)–sarcoplasmic reticulum (SR) dyad junctions, thereby disrupting the close apposition of L-type Ca^2+^ channels (Ca_V_1.2) with ryanodine receptors (RyR2) that trigger SR Ca^2+^ release and myofilament contraction. In a rat ischemic heart failure model expressing low thyroid hormone (TH) function, we used 3D stochastic optical reconstruction microscopy (STORM) to image RyR2 clusters with Ca_V_1.2 channels, and the associated protein junctophilin-2 (Jph2). We tested whether treatment with T3, the biologically active form of TH, throughout progression of the disease would preserve T-tubule structure and dyadic ion channel organization. Confocal microscopy of isolated cardiomyocytes (CMs) stained with ANEPPS membrane dye showed significantly decreased TT density in diseased CMs while T3 treatment attenuated TT disorganization. 3D STORM images of dyadic ion channels labeled with fluorescent-tagged antibodies to RyR-Dylight550, Jph-CF647 and Ca_V_1.2/IgG-Dylight488 were captured. A density-based algorithm defined RyR2 clusters, and a 400 nm spherical 3D volume of interest around each RyR2 cluster’s centroid determined the number of Ca_V_1.2 and Jph2 localizations associated with each RyR2 cluster. Analysis revealed significant reduction in RyR2 cluster size and number with reduced co-localized Jph2 in failing CMs. T3 treatment increased RyR2 cluster numbers and cluster volumes albeit non-significantly, with increased co-clustering of Jph2. The number of Ca_V_1.2 co-localized with RyR2 clusters trended lower in the failing CMs. These results support maintaining TH homeostasis in optimizing the nanoscale organization of Ca^2+^ ion channels in triggering Ca^2+^ release and myofibrillar contraction in patients with heart disease.

## 1. Introduction

Synchronous cardiomyocyte contraction in a normal functioning heart is dependent on intracellular Ca^2+^ transients generated by action potential (AP)-triggered Ca^2+^ influx through sarcolemma L-type Ca^2+^ channels (LTCCs) that in turn triggers Ca^2+^ release from sarcoplasmic reticulum (SR) stores via activated ryanodine receptors (RyR2). This process of Ca^2+^-induced Ca^2+^ release (CICR) necessitates the close apposition of LTCCs with RyR2 clusters localized where junctional SR and transverse (T)-tubule/sarcolemma form dyad structures (reviewed in [1,2]). The nanoscale organization of RyR2 in clusters with co-clustered Ca^2+^ channels at dyads function as coordinated Ca^2+^ release units (CRUs), generating Ca^2+^ sparks leading to myofilament contraction. Recent single-molecule imaging studies reveal that the arrangement of RyR2 in clusters is dynamic and can be altered in diseased hearts, thus affecting CICR [3]. Accessory proteins like the FKBP immunophilins and the phosphorylation of RyR2 have been shown to rearrange RyR tetramer configuration and cluster size, thus affecting Ca^2+^ spark formation [4,5]. The structural dyadic protein junctophilin-2 (Jph2) co-clusters with RyRs and appears capable of forming a complex with individual RyR2 tetramers that can affect cluster size and modulate the Ca^2+^ released [6,7]. Jph2 has also been shown to interact directly with LTCCs and to promote the Ca^2+^ channel’s distribution to dyads where it colocalizes with RyR2 clusters [8]. It has been proposed that the ability of Jph2 to interact with both LTCCs and RyR2 may promote dyad assembly and T-tubule stabilization (reviewed in [9]).

The human heart in end-stage failure undergoes cellular remodeling, including adverse alterations in T-tubule networks and reorganization of dyadic proteins that effectuate excitation–contraction coupling [10,11,12] (reviewed in [13]). Loss of T-tubules in heart failure with reduced ejection fraction (HFrEF) was shown to be triggered by elevated ventricular workload or wall stress, while T-tubule density could be restored by hemodynamic unloading (reviewed in [14]). Subcellular remodeling in HF often mirrors processes observed in the developing heart. As such, T-tubule maturation after birth proceeds with an increasing density of transverse-oriented elements aligned at z-lines where internal RyR2 clusters assemble and form dyadic pairings with LTCCs. This maturation process has been shown to be facilitated by myocyte contractile activity and promoted by glucocorticoids and thyroid hormones [15,16]. Our prior studies using pre-clinical models of heart failure have shown that thyroid hormone (bioactive triiodothyronine, T3) treatment of animals with failing cardiac function restored T-tubule organization and improved cardiomyocyte Ca^2+^ transients and contractile activity, with improved left ventricular hemodynamic measurements [17]. Furthermore, single-molecule localization imaging showed that T3 treatment restored RyR2 clusters in assembly with LTCC and Jph2 proteins along well-organized *z*-lines where dyadic RyR2 cluster localization was anticipated [18,19].

Thyroid hormone (TH) dysfunction is often a hallmark of advanced heart disease in human patients and is associated with worse clinical outcomes, including increased cardiovascular and all-cause mortality [20,21,22,23,24]. Low-T3 Syndrome (LT3S) and subclinical hypothyroidism (SCH) reflect impaired peripheral conversion of thyroxine (T4) to bioactive T3 due to changes in activities of deiodinase (DIO) enzymes that result in reduced T3 content in the diseased myocardium while serum T4 and/or T3 may be within normal ranges [25,26,27]. A recent meta-analysis of 20 randomized controlled trials including 1314 adults (664 patients in the intervention group and 650 in the control group) found that LT3S in HF patients was associated with impaired cardiac function and was a predictor of cardiovascular mortality; patients in the thyroid hormone treatment group showed improvements in endpoints including LV ejection fraction and cardiac output [28]. Current guidelines recognize thyroid dysfunction as prognostically important in HF, and low T3 is viewed as a modifiable pathophysiologic target [29,30]. Emerging consensus recommends thyroid testing in certain HF patients; however, replacement therapy for LT3S in this setting remains individualized, and further guidelines await the outcomes of several active clinical trials [31] (ClinicalTrials.gov NCT05384847, NCT04111536; UK/NIHR.ac.uk).

In support of the clinical implications of utilizing THs as a treatment modality in HF, we have herein used single-molecule localization imaging to ascertain TH’s effects on the nanoscale reorganization of dyadic Ca^2+^ channels in the diseased heart to improve contractile function.

## 2. Results

### 2.1. Physiological Measurements

Animal body weights at the end of the study were not significantly different among the groups with weights (mean ± SD) of 279 ± 13, 280 ± 20 and 278 ± 12 g for the Sham, MI and MI+T3 groups, respectively. Echocardiography recordings of heart rate showed no differences among the three groups, with 303 ± 27, 322 ± 32 and 332 ± 52 bpm for Sham, MI and MI+T3 (Table 1). As expected, cardiac output (CO) was significantly lower (by ~40%) in the MI animals compared to Sham, with an increased output observed in the T3-treated animals, albeit statistically nonsignificant. Ejection fraction decreased significantly from a mean value of 80 ± 7% for Sham animals to 31 ± 10% in MI and 35 ± 14% in MI+T3 animals, indicating heart failure. Increased LV chamber dimensions during systole and diastole, with decreased wall thickness, were indicative of dilated cardiomyopathy. Significantly increased LV posterior wall thickness with T3 treatment relative to untreated MI animals may suggest some improvement in myocardial tissue remodeling. The size of LV infarction was estimated by measuring the area of visible fibrotic tissue after the heart was perfused for myocyte isolation. Animals in which infarction size was estimated to be less than 30% of the LV free wall, and where the echocardiogram measurements showed values outside the standard deviation of the group mean, were not used in the study. The estimated infarct sizes ranged from approximately 30% to 50% of the LV.

### 2.2. T3 and T4 Content in Blood and Ventricular Tissue 

Although immunoassays remain the primary method to measure thyroid hormones and analogs, mass spectrometry has recently been established as a more accurate and sensitive analytical tool for the quantitation of thyroid hormone analytes in different biological fluids and tissues [32].

**Figure 1:**
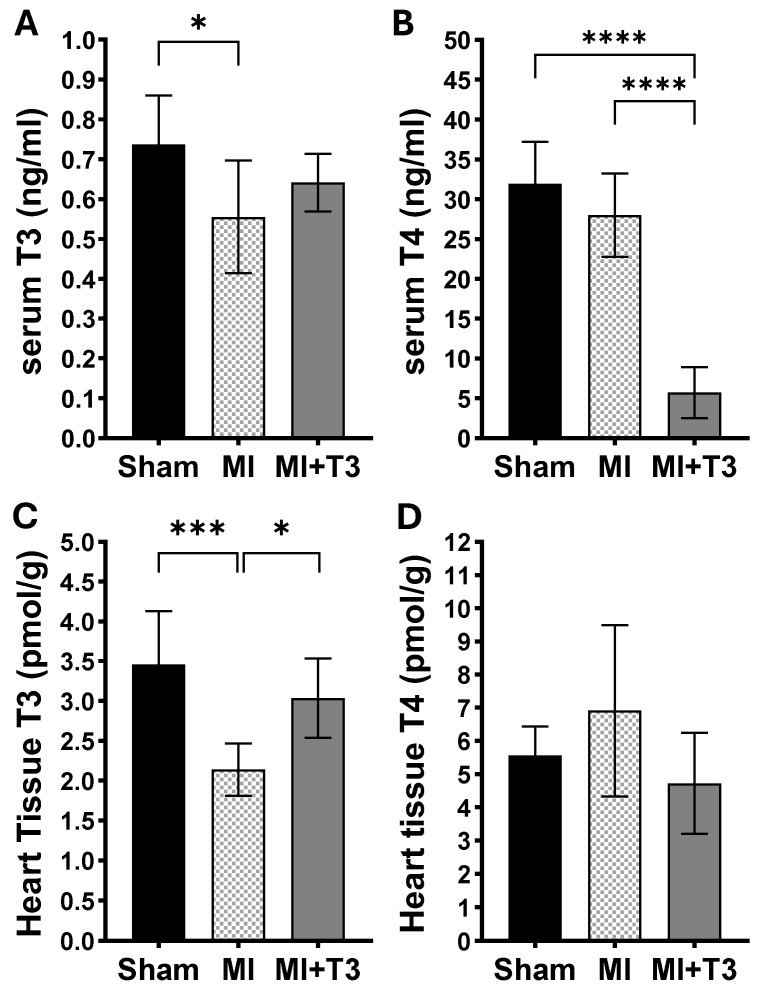
HPLC-MS quantitation of serum and LV tissue T3 and T4 concentrations. Total 3,3′,5 triiodo-L-thyronine (T3) and total 3,5,3′,5′-tetraiodo-L-thyronine (T4) were measured in serum (**A**,**B**) and heart (LV) tissue (**C**,**D**) at the study conclusion. Data are means ± SD, 4 to 8 animals/group. Statistical analysis used one-way ANOVA and post hoc Tukey’s multiple comparisons test. Significance between groups is indicated by brackets; * *p* < 0.05, *** *p* < 0.001, **** *p* < 0.0001.

Mass spectrometric analysis in the present model of MI-induced heart failure showed that both serum T3 concentration and heart tissue T3 content were significantly reduced while T4 concentrations remained within normal ranges, indicating Low-T3 Syndrome (LT3S) (Figure 1). Clinical observational studies have reported LT3S in significant numbers of patients with advanced heart failure [23,24]. In this pre-clinical study, the daily T3 dose used to treat after induction of MI and continued for a four-month period increased serum T3 levels toward normal values (Figure 1A), and importantly, this dose significantly raised heart tissue T3 concentrations to within the normal range (Figure 1C). Furthermore, this low dose of T3 did not increase heart rate, which is a known effect at higher doses. Serum T4 was lowered by T3 treatment, as would be expected from its negative feedback regulation of the hypothalamic–pituitary–thyroid axis, resulting in reduced T4 synthesis and secretion by the thyroid gland (Figure 1B). Notably, heart tissue T4 content was largely unaffected by T3 treatment, suggesting that local control of heart muscle T4 uptake and conversion by deiodinases maintained T4 concentrations (Figure 1D) [26,27].

### 2.3. Transverse-Tubule Organization

**Figure 2:**
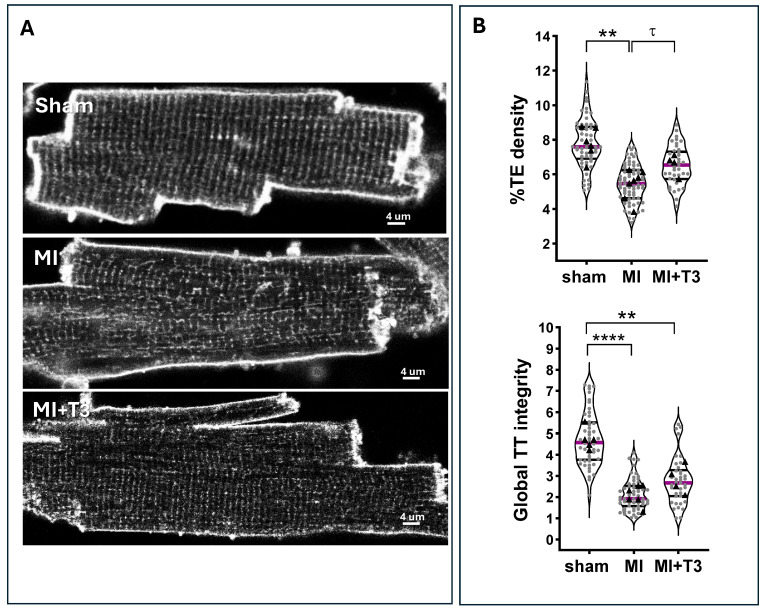
Ventricular myocyte T-tubule (TT) structure analysis. (**A**) Representative confocal images of cardiomyocytes isolated from Sham, MI and MI+T3 study groups that have been stained with membrane-specific di-8-ANEPPS dye. Scale bar, 4 µm. (**B**) AutoTT [33] analysis of the density of transverse-oriented tubule elements (TEs) as percent total tubule elements, and index of global TT integrity (TT*int* = (TE density + LE density) × global TT regularity). Violin plots show the distribution of results of individual cardiomyocytes (grey dots) isolated from 4 to 6 hearts per study group, with the group median (purple line) and quartiles (black lines) shown. The black triangles are the mean value of cells from each heart. Individual cell results of each heart were placed in a subgroup within its study group, and statistical analysis used nested one-way ANOVA with Tukey’s test for multiple group comparisons. Brackets between groups indicate ** *p* < 0.01, **** *p* < 0.0001, ^τ^ *p* = 0.06.

Laser-scanning confocal microscopy images of ANEPPS-stained transverse and longitudinal tubule elements (TEs and LEs) in isolated cardiac myocytes representing hearts from each study group are shown in Figure 2A. Structural analysis of tubule density, regularity and integrity was accomplished using the AutoTT software program developed by Guo and Song [33], and the results are plotted in Figure 2B. Data show that transverse tubule (TE) density was significantly reduced while longitudinal tubules were increased in the hearts of MI rats. Reductions in the global TT integrity measurement that includes assessments of both TE and LE density and regularity points to cardiomyocytes that have disorganized micro-architecture at the dyadic T-tubule/SR junctions that are critical for EC coupling. T3 treatment of rats subjected to MI resulted in cardiomyocytes with an increase in TE density, with other measures of integrity and regularity trending toward control Sham values.

### 2.4. 3D STORM Imaging of RyR2 Clusters and Co-Localizations of Ca_V_1.2 and Jph2

**Figure 3:**
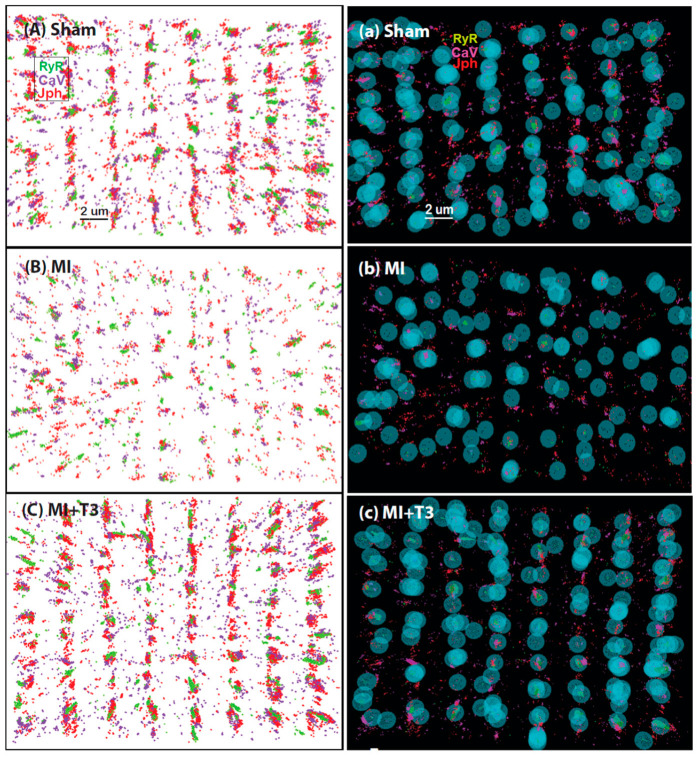
3D STORM imaging and analysis of ventricular myocytes. Panels (**A**) Sham, (**B**) MI, and (**C**) MI+T3 show equivalent regions of interest (ROIs) of representative cells from each study group. Localizations are individual arbitrarily color-coded dots that represent signals in the fluorescent channels corresponding to immunolabeling of RyR2 (green), Ca_V_1.2 (magenta) and Jph2 (red) proteins. Cell images after analysis of localizations in panels (**A**–**C**) are shown in respective panels (**a**–**c**). Cluster analysis of RyR2 signals by DBSCAN (density-based spatial clustering of applications with noise) algorithm shows RyR2 localizations connected by green mesh, and with each cluster encircled by a sphere (blue lattice) with a radius of 400 nm from the RyR2 cluster centroid. Magnification of the image in each panel is the same with xy-axes in the plane of the image, and the z-axis is perpendicular to the image. Scale bar 2 µm applies to all panels.

Figure 3 shows the results of localization and cluster analysis of 3D STORM images of representative cardiomyocytes from each study group. For illustrative purposes, the images in panels (A)–(C) show a small region of a cardiomyocyte spanning eight z-lines (seven sarcomeres) with arbitrarily assigned color-coded individual dots or localizations that represent immunolabeled RyR2 (green), Jph2 (red) and Ca_V_1.2 (magenta) proteins. The X-Y plane is located on the surface of the image with the Z-plane oriented perpendicular to the image surface. Readily apparent is the highly organized alignment of these three proteins into rows that are anticipated to be the locations of sarcomere z-lines or z-discs. This is particularly evident in the Sham (A) and MI+T3 (C) images. The image of the MI cardiomyocyte in panel (B) shows fewer immunolabeled proteins overall, and these appear less well organized at the assumed z-lines. These results are consistent with the T-tubule analysis shown in Figure 2 in which the TE density and regularity at sarcomeric z-lines were significantly reduced in the MI cardiomyocytes. We performed downstream analysis of the fluorescent localizations shown in the images of panels (A)–(C) by algorithmically defining RyR2 clusters by 3D DBSCAN and creating a 400 nm spherical 3D volume of interest around each RyR2 cluster’s centroid. Results show the RyR2 localizations joined by green mesh into clusters, with each RyR2 cluster encircled by a blue sphere (panels (a)–(c)). The highly organized alignment of the RyR2 clusters and surrounding spheres is apparent in the Sham (panel (a)) and MI+T3 (panel (c)) cardiomyocytes but clearly misaligned in the MI cells (panel (b)).

**Figure 4:**
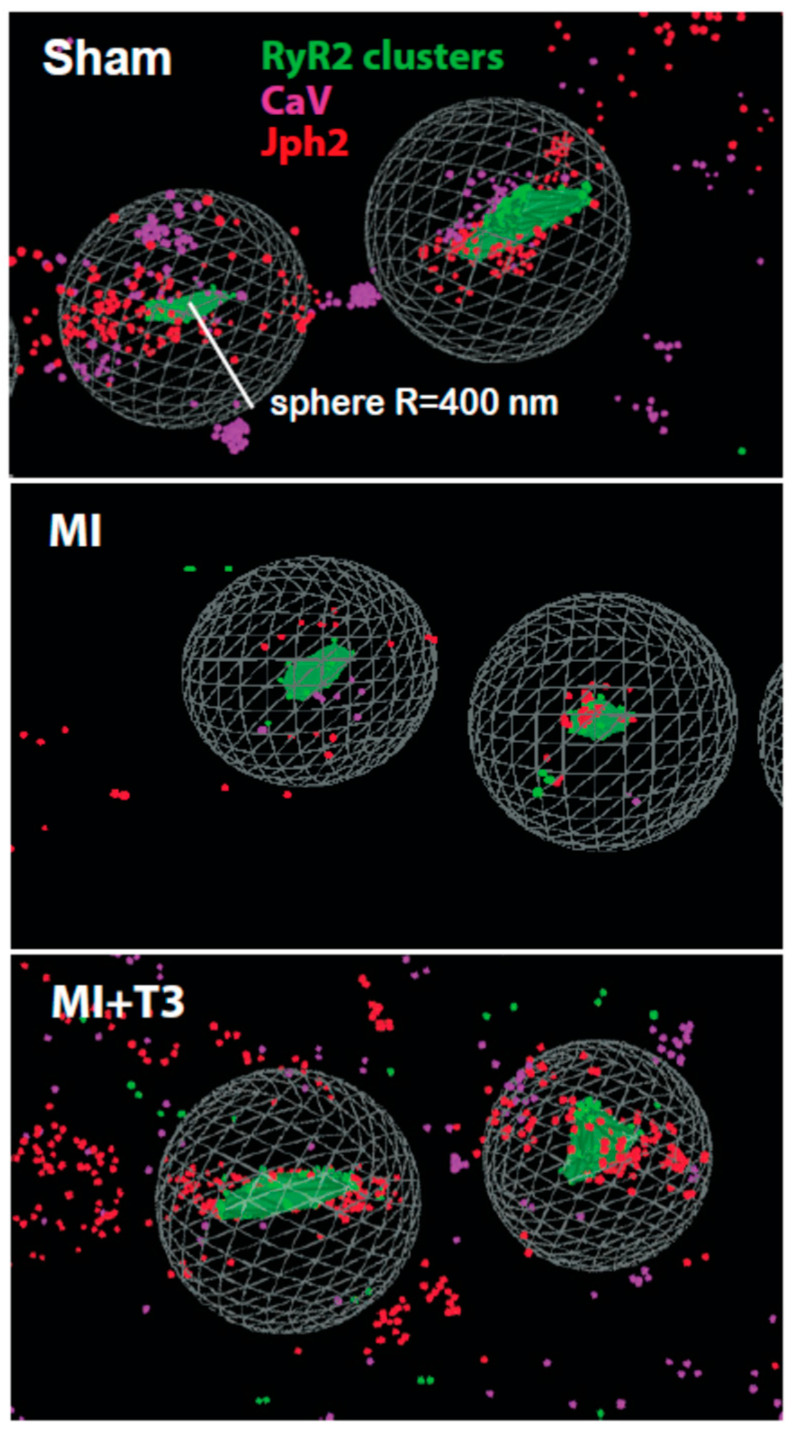
3D STORM images of individual RyR2 clusters. Higher-magnification images of RyR2 clusters from Sham, MI, and MI+T3 cardiomyocytes as shown in Figure 3a–c. RyR2 localizations forming single clusters were identified by DBSCAN algorithm and shown as green dots joined by green mesh. Localizations associated with Ca_V_1.2 (magenta dots) and Jph2 (red dots) were counted within a sphere (lattice) measuring 400 nm radius (R) around the centroid of each RyR2 cluster. Not all localizations reside within the cluster-centered spheres. Reconstruction parameters, imaging conditions and cluster analysis constraints used were the same for images of all sample groups.

Higher magnification of the DBSCAN-analyzed 3D STORM images of cardiomyocytes from each study group showing two adjacent RyR2 clusters encircled by spheres are presented in Figure 4. The RyR2 localizations within each cluster are joined by green mesh with most RyR2 localizations forming clusters at presumed dyads (approximately 70–80% in Sham cardiomyocytes). Some RyR2 localizations did not form clusters, while other RyR2 localizations resided outside the 400 nm cluster radius; this was particularly prevalent in the MI and MI+T3 images. It is well established that LTCCs are juxtaposed to the clusters of RyR2 to enable coordinated calcium release from SR stores during membrane depolarization in EC coupling. In Sham cardiomyocytes (Figure 4), the Ca_V_1.2 localizations (magenta dots) reside in proximity to RyR2 clusters within the 400 nm sphere. Similarly, Jph2 localizations (red dots), a protein known to interact directly with both RyR2 and Ca_V_1.2 channels [7,8], are seen here in close association with RyR2 clusters and Ca_V_1.2. Although significant numbers of Jph2 localizations are located outside the 400 nm spheres, these appear to largely align with presumed z-lines (Figure 3A–C).

**Figure 5:**
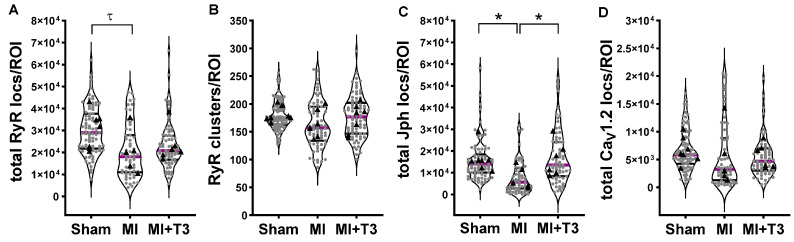
3D STORM image analysis of total RyR2, Jph2 and Ca_V_1.2 per cell. Cell volumes or ROI in 3D longitudinal images of cardiomyocytes measured 3 × 10^11^ nm^3^. Violin plots show the distribution of individual cell results with each group median and quartiles as described in the legend to Figure 2. Individual localization values were averaged from two ROIs of 10–12 cells/heart from 6–7 animals/group. The number of RyR2 clusters per ROI (**B**) and total numbers of RyR2, Jph2 and Ca_V_1.2 localizations (locs) per ROI (**A**,**C**,**D**) were compared among the three study groups (Sham, MI, MI+T3) using nested one-way ANOVA and Tukey’s test for multigroup comparisons, * *p* < 0.05, ^τ^ *p* = 0.06. Violin plots show the distribution of results of individual cardiomyocytes (grey dots) isolated from 4 to 6 hearts per study group, with the group median (purple line) and quartiles (black lines) shown. The black triangles are the mean value of cells from each heart.

Quantitation of all image data represents the average values obtained from two equal ROI volumes per cell. The total RyR2 localization counts (locs) or density within the cell (measured per ROI) were significantly lower in MI than in Sham cardiomyocytes with fewer numbers of RyR2 clusters per ROI (Figure 5A,B). Importantly, T3 treatment of MI hearts was associated with an increased trend in the number of RyR2 locs and clusters per ROI. The total number of Jph2 localizations per ROI was significantly reduced in MI myocytes compared with Sham and increased significantly with T3 treatment (Figure 5C). The total numbers of calcium channels (Ca_V_1.2 localizations) were not significantly different among the three study groups, albeit Ca_V_1.2 numbers trended lower in the MI myocytes (Figure 5D).

**Figure 6:**
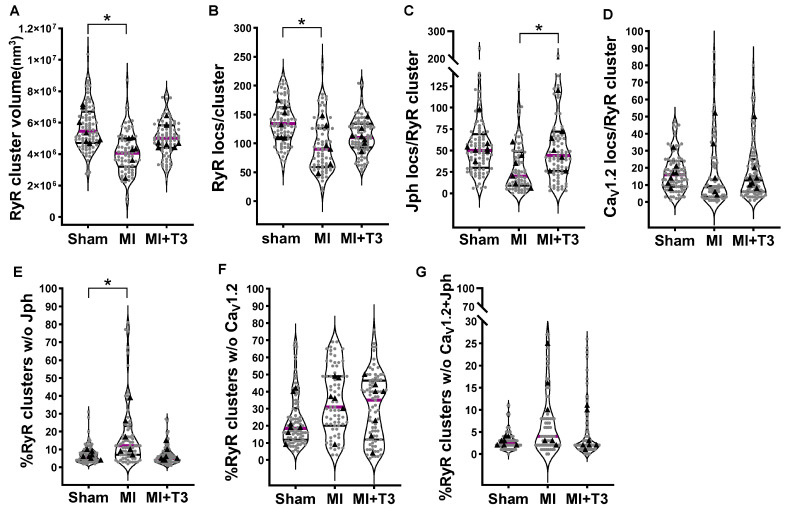
Analysis of RyR2 clusters and associated Jph2 and Ca_V_1.2 in 3D STORM images. The violin plots in each analysis show the distribution of individual cell results (grey dots) with the mean value of each heart (black triangles) in the group and with the group median (purple line) and quartiles (black lines). All individual cell analyses were averaged from two equal ROIs per cell from 10 to 12 cells/heart of 6–7 animals per group. RyR2 clusters were identified by DBSCAN algorithm with subsequent measurements of cluster volume (nm^3^) (**A**) and number of RyR2 localizations per cluster (**B**). Data in (**C**,**D**) are Jph2 and Ca_V_1.2 localizations co-clustered within a sphere measured 400 nm radius of the RyR2 cluster centroid. Results in (**E**–**G**) represent percentage of RyR2 clusters without (w/o) co-clustered Jph2 or Ca_V_1.2 or absent both proteins within the 400 nm sphere. Statistical analysis used nested one-way ANOVA with Tukey’s test for multiple group comparisons: significance at * *p* < 0.05. Violin plots show the distribution of results of individual cardiomyocytes (grey dots) isolated from 4 to 6 hearts per study group, with the group median (purple line) and quartiles (black lines) shown. The black triangles are the mean value of cells from each heart.

Further quantitative analysis revealed that RyR2 cluster volume and the number of RyR2 localizations per cluster decreased significantly in MI myocytes compared to Sham cells (Figure 6A,B) and that T3 treatment tended to increase cluster volume and number. The number of Jph2 proteins localized within a 400 nm sphere of each RyR2 cluster centroid was reduced in the MI cardiomyocytes and was increased to Sham values with T3 treatment (Figure 6C). In contrast, Ca_V_1.2 localizations associated with RyR2 clusters were not statistically different among the groups; however, the median values suggest a decrease in co-clustered Ca_V_1.2 in the MI cardiomyocytes with an increase in the T3-treated MI cells (Figure 6D). We further analyzed the image data for RyR2 clusters that would likely be nonfunctional in EC coupling and found that MI myocytes had significantly more clusters that did not have co-localized Jph2 or did not contain either Ca_V_1.2 or both Jph2 and Ca_V_1.2 proteins (Figure 6E–G). T3-treated MI cardiomyocytes tended to have fewer of these unassociated or “rogue” RyR2 clusters, suggesting that more clusters in the T3-treated hearts were functional in EC coupling than in failing cardiomyocytes.

## 3. Discussion

### 3.1. T-Tubule Structure and Dyadic Ca^2+^ Channel Organization

In the present study we aimed to determine whether thyroid dysfunction (Low-T3 Syndrome, LT3S) in heart failure influenced dyadic ion channel organization. To that end, we used our previously characterized HF model that reproducibly develops LT3S and that could be treated with low-dose T3 for the duration of the progression of disease to specifically interrogate the effects of T3 [17]. In that prior HF study, T3 treatment improved cardiomyocyte calcium transients and contractile dynamics and increased T-tubule density and regularity, suggesting enhanced EC coupling. Furthermore, using a hypothyroid disease model we showed that T3 treatment rescued the nanoscale defects in dyadic ion channel organization in the thyroid hormone-deficient cardiomyocytes [19]. Those data also showed near-complete restoration of normal structural organization of T-tubules that aligned with Ca^2+^ spark recordings. Advances in microscopy have been instrumental in showing adverse remodeling of T-tubule networks in failing human hearts and in animal models of HF in which disarray of dyad structures results in loss of synchronous Ca^2+^ release leading to impaired EC coupling [11,34,35,36] (reviewed in [1]).

The MI-induced heart failure animal model has been shown to result in myocardial tissue remodeling in which cardiomyocytes that border the infarct area experience greater stress and hemodynamic load than myocytes distal to the fibrotic infarct. We therefore expected that viable cardiomyocytes isolated from the LV free wall of MI hearts would experience greater variations in the extent of remodeling than myocytes isolated from Sham hearts. As such, the violin plots illustrate the wide distribution of cell imaging results regardless of treatment. Despite these variations, T-tubule organization and approximation of RyR2 clusters with Ca_V_1.2 and Jph2 proteins showed significant disarray and reductions in the failing cardiomyocytes with a consistent trend toward improvement as a result of T3 treatment during the disease progression. Tissue remodeling after acute MI injury involves a complex biological response such that any single factor would be unlikely to completely reverse the process; however, these study results provide evidence that treating the low-T3 condition that presents after myocardial injury may provide benefit.

We had previously shown that T3 treatment following acute MI improved Ca^2+^ transients and contractile dynamics in the individual isolated ventricular myocyte, associated with significant increases in cardiac hemodynamics as measured by rates of LV pressure development in systole and diastole, and in increased cardiac ejection fraction. To interrogate whether ion channel organization in dyads in response to T3 would enhance Ca^2+^-induced Ca^2+^ release, high-speed imaging of Ca^2+^ sparks in live cells concurrent with RyR2 localization microscopy would be required as published by Hou et al. [3]. These authors showed that failing cardiomyocytes exhibited more multi-release Ca^2+^ sparks that were larger and propagated further than single-release sparks, and that these sparks initiated at closely spaced, high-density RyR2 clusters. STORM imaging revealed dispersion of RyR2 clusters into smaller cluster sizes of higher density in the failing myocytes. In support of those observations, our STORM imaging analysis revealed smaller RyR2 cluster volumes in failing cardiomyocytes, and cluster densities (RyR2 localizations/cluster) that were proportionately decreased compared to myocytes from Sham or from T3-treated hearts (Figure 6A,B). Our data also showed fewer total RyR2 localizations per cell (ROI), and fewer clusters (Figure 5A,B). STORM image analysis of failing cardiomyocytes revealed a trend to fewer Ca_V_1.2 localizations associated with RyR2 clusters and more clusters without co-localized Ca^2+^ channels, essentially rendering these clusters nonfunctional in EC coupling. In addition to changes in configuration of RyR2 tetramers in clusters, the disarray of clusters and LTCC at jSR-TT dyads along z-lines in failing myocytes would further impact Ca^2+^ spark generation and Ca^2+^ efflux. Confocal and STORM imaging data revealed that T3 treatment during the progression of HF largely attenuated adverse remodeling of T-tubules and preserved the arrangement of RyR2 clusters and Jph2 and Ca_V_1.2 localizations in proximity to the RyR2 clusters. Clearly, to equate the nanoscale changes in Ca^2+^ channel clustering and organization of the SR-TT dyad structures in heart failure to in vivo measures of hemodynamic function may be inherently difficult, particularly in an ischemia model of HF with significant tissue injury and fibrosis. However, advanced imaging of Ca^2+^ sparks in live cells concurrent with RyR2 localization microscopy as described by Hou et al. [3] would provide evidence of intracellular structure normalization in advancing organ-level functional recovery.

### 3.2. Nanoscale Mechanisms of Thyroid Hormone (TH) Action

Thyroid hormones (bioactive T3) have long been known to increase cardiomyocyte contractile function by augmenting Ca^2+^ re-uptake into the SR by increasing SERCA2 (SR Ca-ATPase) and diminishing PLN (phospholamban)-inhibitory activities [37]. Other cellular targets of T3 include mitochondria, myofilament proteins and membrane ion currents with effects on bioenergetics, protein turnover and metabolic homeostasis (reviewed in [20,21,22]). Thus, thyroid dysfunction with system-wide loss of homeostasis would inevitably adversely affect cardiac function.

Evidence that THs have effects on the volume and surface area of T-tubules and terminal cisternae in myocytes was first reported in 1986 [38]. Recent studies of neonatal cardiomyocytes, organoids and engineered myocardium have shown that maturation of T-tubule networks requires thyroid hormones [15,16,39]. Nanoscale imaging analyses have further advanced our understanding of TH’s role in the organization of ion channels at T-tubule/jSR dyads, and thus establish a critical role of T3 in EC coupling. The developmental maturation of cell membranes with integral membrane proteins and ion channels involves complex processes and effectors [40]. The mechanisms by which T3 potentially influences these processes are largely unknown, although junctophilin (Jph) proteins that provide structural integrity to dyad structures in muscle cells may be targets of T3 regulation. We have reported that Jph2 expression is upregulated by T3 in cardiomyocytes; however, no known genomic T3 response elements have been identified, although microRNAs may play a role [17,41]. Our nanoscale imaging studies in failing cardiomyocytes and in hypothyroid hearts have shown that Jph2 consistently co-clusters with RyR2 and is highly responsive to T3 treatment [18]. The role of Jph proteins in neuronal and myocyte excitability has recently been reviewed in detail [9]. Reduction in Jph2 in patients with heart failure has been shown to reduce T-tubule/jSR complexes, causing dysfunction in Ca^2+^ handling [12,42]. Considering that heart failure is a chronic degenerative disease with a high probability of co-existent thyroid dysfunction, it is imperative that consideration be given to evaluating thyroid hormone status as a risk factor in this population with the potential of TH treatment options [30,43,44].

### 3.3. Study Limitations

We have used three-color 3D STORM to image RyR2, Ca_V_1.2 and Jph2 proteins localized at dyad structures which are integral to CICR in cardiomyocytes. The spatial resolution of fluorescent-tagged antibodies bound to these proteins does not permit determination of individual RyR2 tetrameric channels, nor does it provide an absolute number of channels. Since the binding affinities of different antibodies to their target proteins can vary greatly [6], we have designated the fluorescence signal as “localization” rather than “protein or channel” numbers. Additionally, Ca_V_1.2 was detected using a secondary IgG antibody conjugated to a fluorescent probe (DyLight488) whereas RyR2 and Jph2 were detected using fluorescently conjugated primary antibodies. The use of fluorescent secondary antibodies vs. primary antibodies could result in variable labeling efficiency, and in reduced detection power in the 488 nm channel identifying Ca_V_1.2 during image capture. Despite these technical limitations, we posit that the study results and conclusions are valid since our analyses are viewed as comparisons of treatment groups to each other and relative to controls.

As with our prior studies, we used adult female rats in this heart failure model because female body weights plateau after approximately 12 weeks of age, and therefore any cardiac remodeling or hypertrophy occurring after myocardial infarction would be independent of changes in body mass. That said, the clinical significance of assessing thyroid dysfunction in HF is applicable to both sexes and the physiological responses may likely be sex-specific and will require additional study.

### 3.4. Highlights

In a pre-clinical model of heart failure that recapitulated Low-T3 Syndrome, we showed that T3 treatment early in disease progression preserved T-tubule networks and dyadic ion channel organization.

## 4. Materials and Methods

### 4.1. Animal Model and Treatment Protocols

Study protocols were approved by the Institutional Animal Care and Use Committee of the New York Institute of Technology College of Osteopathic Medicine. Animals were treated in accordance with the National Institutes of Health Guidelines for the Use and Care of Laboratory Animals (HHS Pub. No.85-23). Female Sprague-Dawley rats (Envigo RMS, Inc., Indianapolis, IN, USA) aged 14 to 18 weeks, weighing 244 ± 12 g, underwent thoracotomy with permanent ligation of the left anterior descending coronary artery (infarction, MI) or sham surgery without vessel ligation. Twenty hours after surgery, surviving MI animals were randomized to treatment with triiodo-L-thyronine (T3, 5 μg/kg/d; Sigma Aldrich, St. Louis, MO, USA) (MI+T3 group) or vehicle (MI group) in drinking water as previously described [17]. Sham animals received untreated tap water. Rats were housed under controlled temperature conditions with 12 h light/dark cycles and access to water and standard rat chow ad libitum. The study was terminated after 16 to 18 weeks of treatment. Hearts from 9 to 12 animals in each study group were used for cardiomyocyte (CM) isolation and imaging. Heart tissues from separate groups of 6 to 9 animals were used for T3 and T4 analysis. Animal numbers for each analysis are indicated in the text.

### 4.2. Echocardiography

A Vevo 3100 (FUJIFILM VisualSonics, Inc., Toronto, ON, Canada) ultrasound imaging system coupled with a 25 MHz transducer probe was used to record LV chamber dimensions as previously described [45]. Briefly, at the end of the treatment period, rats were lightly anesthetized with 1.5% isoflurane to obtain two-dimensional echocardiograms in M-mode from LV short-axis and long-axis views. The Vevo Lab analysis tool was used to measure LV wall thickness and chamber diameter in diastole and systole to determine LV function and mass.

### 4.3. T3 and T4 Quantitation in Heart Tissue and Serum

Following echocardiography, fully anesthetized rats underwent left thoracotomy to expose the heart for blood collection from the RV, and then the heart was flushed with ice-cold EDTA-containing buffer, removed from the chest and either retrograde-perfused for enzymatic isolation of viable LV myocytes for microscopy or rapidly frozen in liquid nitrogen for later analysis of T3 and T4. Tissue and serum samples were stored at −80 °C until analysis. Serum and LV samples from the same animals were analyzed for T4 and T3 using 6 to 9 animals per study group.

#### 4.3.1. LC-MS Methodology: Sample Preparation

Serum samples were processed for quantification of total T_3_ and T_4_ as previously published [32]. Unlabeled 3,3′,5-Triiodo-L-thyronine (T_3_), 3,3′,5′-Triiodo-L-thyronine (rT_3_), L-Thyroxine (T_4_), ^13^C_6_-rT_3_/T_3_ and ^13^C_6_-T_4_ were obtained as Certified Reference Material from Cerilliant^®^ (Round Rock, TX, USA) (100 μg/mL in 0.1 N NH_3_ in Methanol). Methanol, acetonitrile, water, and isopropanol (Thermo Fisher Scientific, Waltham, MA, USA) were at MS-grade purity. A stable isotope-labelled internal standard mixture (10 µL of a 100 ng/mL (^13^C_6_-rT_3_/T_3_ and ^13^C_6_-T_4_) was added to 100 µL of serum. Samples were vortexed and kept at room temperature for 30 min, followed by addition of 300 μL of cold acetone to allow protein precipitation. After centrifugation (22,780× *g* for 10 min), the supernatants were collected and concentrated to ~100 µL by vacuum centrifugation. After addition of 400 μL of 0.1 M potassium acetate buffer (pH = 4), samples underwent Solid Phase Extraction (SPE) using Agilent Bond-Elut Certify 130 mg SPE cartridges (Agilent Technologies Inc., Santa Clara, CA, USA). Final sample eluates were vacuum-dried, then reconstituted with 100 μL of methanol/water (30/70, *v*/*v*), and 5 µL was injected into the LC-MS system. Calibration curves were prepared by serially diluting the standards in neat methanol at 0.10–0.25–0.50–1.00–2.50–5–10–25–50–100 ng/mL. At the end of the procedure, both calibration points and samples had the same nominal concentration of internal standard.

LV tissue samples (50–100 mg), kept at −80 °C, were quickly transferred to 2 mL homogenizing PRECELLYS^®^ tubes, suspended in 1 mL of a solution made of 85% aqueous acetonitrile containing 0.2 ng/mL internal standard mixture (^13^C_6_-rT_3_/T_3_ and ^13^C_6_-T_4_) and processed as previously reported [46,47]. Briefly, samples were vortexed, sonicated for 15 min and then homogenized using a Precellys^®^24-Dual Homogenizer (Bertin Technologies, Montigny-le-Bretonneux, France) through three homogenization steps at 4 °C, of 45 s with 60 s pause at 8000 rpm. Homogenized samples were sonicated again for 15 min, then centrifuged for 15 min at 22,780× *g* and supernatants centrifuged again before drying by vacuum centrifugation. Samples were reconstituted using 100 µL of methanol/water (30:70), vortexed for 10 min, centrifuged and then transferred into autosampler vials from which 10 µL were injected into the LC-MS system. The quantification of analytes was performed using standard T_3_ and T_4_ calibration curves in methanol from 0.025 ng/mL to 5 ng/mL, such that both calibration points and samples had the same nominal concentration of internal standard.

#### 4.3.2. LC-MS Configuration

The instrumental layout consisted in a Vanquish Horizon UHPLC System (Thermo Fisher Scientific) including a binary pump, a column oven set at 20 °C and a thermostated autosampler (set at 15 °C), coupled to a Thermo Scientific Q Exactive HF Orbitrap LC-MS/MS System, equipped with a HESI source. Chromatographic separation was achieved using a 2.1 × 50 mm, 3.5 μm particle size, Waters Xselect HSS T3 column (Milford, MA, USA). Mobile phase A consisted of 0.1% FA in water, and mobile phase B of MeOH/ACN (20/80 by volume) containing 0.1% FA. Gradient elution (400 μL/min flow rate) was performed as follows: 0–1 min (B) 20%, 5 min (B) 70%, 5.1–6 min (B) 100%, 6.1–6.9 min (B) 20%, 7–8 min (B) 20% at 800 μL/min, 8–8.5 min (B) 20%. Eluates were ionized using an in-line HESI source kept at +5.5 kV compared to the MS inlet (sheath gas = 60 a.u.; sweep gas = 10 a.u.; capillary temperature = 400 °C; auxiliary gas heater temperature = 400 °C). The mass spectrometer was operated in selected ion monitoring (tSIM) mode with targeted ions specified in an inclusion list covering all the analytes of interest: T3/rT3 (651.8 *m*/*z*), T3/rT3 ^13^C_6_ (657.8 *m*/*z*), T4 (777.7 *m*/*z*) and T4 ^13^C_6_ (783.7 *m*/*z*). After quadrupolar isolation of the ions of interest (isolation window = 1.6 *m*/*z*; RF lens = 30%), up to 1 × 10^6^ charges were accumulated (max injection time = 118 ms), and ions were recorded using the orbitrap detector at 60,000 resolution (at *m*/*z* 200) in profile mode. System control and data acquisition were performed using Thermo Scientific SII Xcalibur V. 4.4.16.14. Integration of area under the curve for extracted ion chromatograms for quantitative analysis was performed using Skyline v.22.2.0.527.

### 4.4. Cardiomyocyte Isolation and Confocal Imaging of T-Tubules

Left ventricular myocytes were isolated as previously described [18]. Briefly, after collagenase digestion of the heart, the infarcted LV tissue was separated from the remainder of the LV to measure size of the infarct area. The non-infarcted LV free wall was then used to isolate viable cardiomyocytes that were plated onto laminin-coated glass-bottom eight-well chamber slides (Ibidi) (Thermo Fisher Scientific) in M199 medium containing 10% FBS and 2,3-butanedione monoxime (BDM, 10 mM) and allowed to adhere for 2 h. Adherent cardiomyocytes (CMs) were labeled with di-8-ANEPPS (5 μM in HBSS containing 10 mM BDM; Biotium Inc., Fremont, CA, USA) for 20 min. at 37 °C. Live myocytes (10–12 CMs per heart) were imaged using the Axio Observer.Z1/7 Zeiss 980 LSM microscope with Plan-Apochromat 63x/1.40 Oil DIC M27 objective (Carl Zeiss Microscopy LLC, White Plains, NY, USA). Nine images captured at 3.2 um intervals of each cell were obtained and transverse- and longitudinal-oriented elements (TE, LE) were analyzed for density and integrity using an automated computational program (AutoTT) developed by Guo and Song [33].

### 4.5. Immunofluorescence Staining for Three-Color 3D STORM

Cardiomyocytes isolated on the same day from different study groups were plated onto eight-well chamber slides, and then adherent cells were fixed in 4% paraformaldehyde prior to permeabilization and blocking with 0.2% Triton X-100 and 5% goat serum in PBS for 1 h [19]. CMs were first incubated in 0.02% TX-100/5% goat serum with anti-Ca_V_1.2 (CACNA1C) rabbit polyclonal antibodies (1:400; ACC-003; Alomone Labs, Jerusalem, Israel) overnight at 4 °C, followed the next day by goat anti-rabbit IgG(H+L) DyLight^®^488 (1:2000; ab96883; Abcam, Cambridge, MA, USA) for 1 h at room temperature. After appropriate washes with PBS, cells were briefly fixed with 4% PFA, then washed and incubated overnight at 4 °C with two other primary antibodies: anti-RyR2 mouse monoclonal antibody-DyLight^®^ 550 (C3-33) (1:200; NBP2-80143R; Novus Biologicals, Centennial, CO, USA) and anti-Jph2 rabbit polyclonal antibody (1:200; 405300 Invitrogen; Thermo Fisher Scientific) labeled with CF^®^647 using a Biotium Mix-n-Stain™ STORM dye antibody labeling kit (cat no. 92554, Biotium Inc.). Following PBS washes, labeled slides were stored in sodium-azide/PBS at 4 °C until they were imaged.

### 4.6. STORM Image Capture, Analysis and Data Computation

Three-dimensional 3D STORM images were captured using the Nanoimager-S super-resolution microscope (Oxford Nanoimaging Ltd. (ONI), Oxford, UK). As we have previously detailed [19], color channel mapping was calibrated using 200 nm diameter Tetraspek beads, and a cylindrical lens was placed in the light path to create astigmatic distortion of the Gaussian spots that depends on the distance from the focal plane of each bead along the *z*-axis to create a three-dimensional map covering the field of view. Antibody-labeled CMs in the 8-well chamber slides were incubated in a blinking buffer (BCubed buffer, ONI) during STORM imaging with a fresh buffer change every 30 min. Single-molecule localization data from the excitation of each fluorophore were recorded sequentially at 640 nm, 561 nm and 488 nm to avoid cross-excitation among channels. Each image was acquired as a series of 5000 raw frames at 50 ms/frame at each wavelength. The focal plane used for image capture was determined by visual inspection as the brightest fluorescence signal in the 561 nm channel localizing RyR2 proteins.

Raw image data were corrected for drift and filtered according to a standard protocol integrated into ONI imaging software that we have previously described [19]. The filtered image data were exported from the Nanoimager NimOS software v.1.19 and loaded into the LumeVR software program for localization microscopy data analysis (LumeVR, Inc., Oxford, UK). Two identical regions of interest (ROI = 3.0 × 10^11^ nm^3^) were delineated within each cell image away from the cell periphery or nuclei. Clustering of RyR2 localizations (excitations captured in the 561 nm channel) was accomplished using a density-based spatial clustering of applications with noise (DBSCAN) algorithm [48] with a RyR2 localization neighbor search radius of 100 nm. The minimum number of localizations that defined an RyR2 cluster was set to 4, with maximum localizations per cluster of 4000. The RyR2 cluster size limits were set to a minimum of 50 nm and a maximum of 2000 nm. The numbers of localizations of Jph2 and Ca_V_1.2 within a sphere of 400 nm radius from the centroid of each RyR2 cluster were recorded. Final data analysis from each ROI consisted of RyR2 cluster volume, number of RyR2 localizations per cluster, numbers of Jph2 and Ca_V_1.2 localizations within a sphere of 400 nm of the RyR2 cluster centroid, and total RyR2, Jph2 and Ca_V_1.2 localizations per ROI. Values obtained from the two ROIs analyzed per cell were averaged from a total of 10–12 cells per heart, and results are expressed as mean values per animal or using individual cell data from 6–9 hearts per study group.

### 4.7. Statistical Analyses

Description of data analyses and presentation are detailed in the figure legends or table. Data were assessed for normality distribution and equal variance, and statistical significance among groups was determined by one-way ANOVA, and post hoc pairwise comparisons of group mean used Tukey’s multiple comparisons test (Prism software v10.6.1; GraphPad Prism Software, Inc., San Diego, CA, USA). Data are presented as study group means ± SD. Statistical significance was set at *p* < 0.05.

All confocal and STORM imaging data are shown as violin plots to illustrate the distribution of results of individual cardiomyocytes from every animal in each study group, and the median value per group with quartiles is indicated. For statistical analysis, individual cell results from each heart were placed into subgroups within its appropriate study group (Sham, MI, MI+T3), and statistical significance among the groups was determined by nested one-way ANOVA and Tukey’s test was used for multiple group comparisons. The level of significance was set at 5% unless stated otherwise (these analyses and data presentation used Prism software v10.6.1).

## Figures and Tables

**Table 1:** Echocardiography measurements.

	Sham (n = 12)	MI (n = 10)	MI + T3 (n = 9)
Heart rate (bpm)	303 ± 27	322 ± 32	332 ± 52
Cardiac output (mL/min)	56 ± 9	40 ± 15 ^a^	52 ± 16
Fractional shortening %	51 ± 5	15 ± 6 ^d^	19 ± 8 ^d^
Ejection fraction %	80 ± 7	31 ± 10 ^d^	35 ± 14 ^d^
LVDs (mm)	3.304 ± 0.455	8.095 ± 1.233 ^d^	7.816 ± 1.626 ^d^
LVDd (mm)	6.691 ± 0.456	9.529 ± 1.186 ^d^	9.358 ± 1.300 ^d^
LV end systolic vol (μL)	46 ± 14	363 ± 116 ^d^	343 ± 153 ^d^
LV end diastolic vol (μL)	233 ± 36	517 ± 137 ^d^	499 ± 160 ^d^
LVAWs (mm)	2.787 ± 0.505	0.944 ± 0.448 ^d^	1.148 ± 0.505 ^d^
LVAWd (mm)	1.697 ± 0.344	1.019 ± 0.469 ^b^	1.106 ± 0.548 ^a^
LVPWs (mm)	2.946 ± 0.283	2.168 ± 0.360 ^c^	2.639 ± 0.499 ^e^
LVPWd (mm)	1.676 ± 0.249	1.382 ± 0.400	1.861 ± 0.437 ^e^

Myocardial infarction-induced animals receiving oral vehicle (MI) or T3 (triiodothyronine) (MI+T3) for 4 months post-MI surgery. Sham, surgical Sham animals. Left ventricle (LV) chamber diameter in end diastole (LVDd) or systole (LVDs); LV anterior wall (LVAW) or posterior wall (LVPW). Values are mean ± SD. Statistical analysis used one-way ANOVA with Tukey’s test for multigroup comparisons. n = animals/group. ^a^ *p* < 0.05 vs. Sham, ^b^ *p* < 0.01 vs. Sham, ^c^ *p* < 0.001 vs. Sham, ^d^ *p* < 0.0001 vs. sham, ^e^ *p* < 0.05 vs. MI.

## Data Availability

The data generated or analyzed for this study are available from the corresponding authors upon reasonable request.

## Abbreviations

ANEPPS	aminonaphthyl–ethenylpyridinium
BDM	2,3-butanedione monoxime
Ca_V_1.2	L-type calcium channel voltage-gated subunit 1.2
CICR	calcium-induced calcium release
CM	cardiomyocyte
CRU	calcium release unit
DIO	(thyroid) deiodinases
ECC	excitation–contraction coupling
FKBP	cytoplasmic receptor that binds immunosuppressant drugs, i.e., FK506, rapamycin
HF	heart failure
HFrEF	heart failure with reduced ejection fraction
HFpEF	heart failure with preserved ejection fraction
Jph2	junctophilin-2
HBSS	Hank’s balanced salt solution
LC-MS	liquid chromatography–mass spectrometry
LEs	longitudinal tubule elements
LT3S	low-T3 syndrome
LTCC	L-type calcium channel
LVEF	left ventricular ejection fraction
MI	myocardial infarction
ONI	Oxford Nanoimaging Ltd.
PLN	phospholamban
ROI	region of interest
RV	right ventricle
RyR2	ryanodine receptor-type 2
SR	sarco(endo)plasmic reticulum
SERCA2	sarco(endo)plasmic reticulum calcium ATPase
STORM	stochastic optical reconstruction microscopy
SCH	subclinical hypothyroidism
T3	3,5,3′-triiodo-L-iodothyronine
T4	thyroxine (3,5,3′,5′-tetraiodo-L-thyronine)
TEs	transverse tubule elements
THs	thyroid hormones
TTs	transverse tubules

## References

[1] Louch W.E., Perdreau-Dahl H., Edwards A.G. (2022). Image-Driven Modeling of Nanoscopic Cardiac Function: Where Have We Come From, and Where Are We Going?. Front. Physiol..

[2] Scriven D.R., Asghari P., Moore E.D. (2013). Microarchitecture of the dyad. Cardiovasc. Res..

[3] Hou Y., Laasmaa M., Li J., Shen X., Manfra O., Norden E.S., Le C., Zhang L., Sjaastad I., Jones P.P. (2023). Live-cell photo-activated localization microscopy correlates nanoscale ryanodine receptor configuration to calcium sparks in cardiomyocytes. Nat. Cardiovasc. Res..

[4] Asghari P., Scriven D.R., Ng M., Panwar P., Chou K.C., van Petegem F., Moore E.D. (2020). Cardiac ryanodine receptor distribution is dynamic and changed by auxiliary proteins and post-translational modification. elife.

[5] Asghari P., Scriven D.R.L., Shahrasebi S., Valdivia H.H., Alsina K.M., Valdivia C.R., Navarro-Garcia J.A., Wehrens X.H.T., Moore E.D.W. (2024). Phosphorylation of RyR2 simultaneously expands the dyad and rearranges the tetramers. J. Gen. Physiol..

[6] Jayasinghe I., Clowsley A.H., Lin R., Lutz T., Harrison C., Green E., Baddeley D., Di Michele L., Soeller C. (2018). True Molecular Scale Visualization of Variable Clustering Properties of Ryanodine Receptors. Cell Rep..

[7] Munro M.L., Jayasinghe I.D., Wang Q., Quick A., Wang W., Baddeley D., Wehrens X.H., Soeller C. (2016). Junctophilin-2 in the nanoscale organisation and functional signalling of ryanodine receptor clusters in cardiomyocytes. J. Cell Sci..

[8] Gross P., Johnson J., Romero C.M., Eaton D.M., Poulet C., Sanchez-Alonso J., Lucarelli C., Ross J., Gibb A.A., Garbincius J.F. (2021). Interaction of the Joining Region in Junctophilin-2 with the L-Type Ca(2+) Channel Is Pivotal for Cardiac Dyad Assembly and Intracellular Ca(2+) Dynamics. Circ. Res..

[9] Lehnart S.E., Wehrens X.H.T. (2022). The role of junctophilin proteins in cellular function. Physiol. Rev..

[10] Pinali C., Bennett H., Davenport J.B., Trafford A.W., Kitmitto A. (2013). Three-dimensional reconstruction of cardiac sarcoplasmic reticulum reveals a continuous network linking transverse-tubules: This organization is perturbed in heart failure. Circ. Res..

[11] Seidel T., Navankasattusas S., Ahmad A., Diakos N.A., Xu W.D., Tristani-Firouzi M., Bonios M.J., Taleb I., Li D.Y., Selzman C.H. (2017). Sheet-Like Remodeling of the Transverse Tubular System in Human Heart Failure Impairs Excitation-Contraction Coupling and Functional Recovery by Mechanical Unloading. Circulation.

[12] Hou Y., Bai J., Shen X., de Langen O., Li A., Lal S., Dos Remedios C.G., Baddeley D., Ruygrok P.N., Soeller C. (2021). Nanoscale Organisation of Ryanodine Receptors and Junctophilin-2 in the Failing Human Heart. Front. Physiol..

[13] Dibb K.M., Louch W.E., Trafford A.W. (2022). Cardiac Transverse Tubules in Physiology and Heart Failure. Annu. Rev. Physiol..

[14] Ibrahim M., Terracciano C.M. (2013). Reversibility of T-tubule remodelling in heart failure: Mechanical load as a dynamic regulator of the T-tubules. Cardiovasc. Res..

[15] Parikh S.S., Blackwell D.J., Gomez-Hurtado N., Frisk M., Wang L., Kim K., Dahl C.P., Fiane A., Tonnessen T., Kryshtal D.O. (2017). Thyroid and Glucocorticoid Hormones Promote Functional T-Tubule Development in Human-Induced Pluripotent Stem Cell-Derived Cardiomyocytes. Circ. Res..

[16] Jackman C., Li H., Bursac N. (2018). Long-term contractile activity and thyroid hormone supplementation produce engineered rat myocardium with adult-like structure and function. Acta Biomater..

[17] An S., Gilani N., Huang Y., Muncan A., Zhang Y., Tang Y.D., Gerdes A.M., Ojamaa K. (2019). Adverse transverse-tubule remodeling in a rat model of heart failure is attenuated with low-dose triiodothyronine treatment. Mol. Med..

[18] Gilani N., Wang K., Muncan A., Peter J., An S., Bhatti S., Pandya K., Zhang Y., Tang Y.D., Gerdes A.M. (2021). Triiodothyronine maintains cardiac transverse-tubule structure and function. J. Mol. Cell. Cardiol..

[19] Charest A., Nasta N., Siddiqui S., Menkes S., Thomas A., Saad D., Forman J., Huang X., Sison C.P., Gerdes A.M. (2024). Nanoscale organization of cardiac calcium channels is dependent on thyroid hormone status. Am. J. Physiol..

[20] Jabbar A., Pingitore A., Pearce S.H., Zaman A., Iervasi G., Razvi S. (2017). Thyroid hormones and cardiovascular disease. Nat. Rev. Cardiol..

[21] Klein I., Ojamaa K. (2001). Thyroid hormone and the cardiovascular system. N. Engl. J. Med..

[22] Gerdes A.M., Ojamaa K. (2016). Thyroid Hormone and Cardioprotection. Compr. Physiol..

[23] Iervasi G., Pingitore A., Landi P., Raciti M., Ripoli A., Scarlattini M., L’Abbate A., Donato L. (2003). Low-T3 syndrome: A strong prognostic predictor of death in patients with heart disease. Circulation.

[24] Zhou P., Huang L., Zhai M., Huang Y., Zhuang X., Liu H., Zhang Y., Zhang J. (2023). Prognostic role and relationship of thyroid dysfunction and lipid profile in hospitalized heart failure patients. Clin. Cardiol..

[25] Wassen F.W., Schiel A.E., Kuiper G.G., Kaptein E., Bakker O., Visser T.J., Simonides W.S. (2002). Induction of thyroid hormone-degrading deiodinase in cardiac hypertrophy and failure. Endocrinology.

[26] Sabatino L., Vassalle C., Del Seppia C., Iervasi G. (2021). Deiodinases and the Three Types of Thyroid Hormone Deiodination Reactions. Endocrinol. Metab..

[27] Simonides W., Tijsma A., Boelen A., Jongejan R., de Rijke Y., Peeters R., Dentice M., Salvatore D., Muller A. (2023). Divergent Thyroid Hormone Levels in Plasma and Left Ventricle of the Heart in Compensated and Decompensated Cardiac Hypertrophy Induced by Chronic Adrenergic Stimulation in Mice. Metabolites.

[28] Shi C., Bao Y., Chen X., Tian L. (2022). The Effectiveness of Thyroid Hormone Replacement Therapy on Heart Failure and Low-Triiodothyronine Syndrome: An Updated Systematic Review and Meta-analysis of Randomized Controlled Trials. Endocr. Pract..

[29] Xu Y., Derakhshan A., Hysaj O., Wildisen L., Ittermann T., Pingitore A., Abolhassani N., Medici M., Kiemeney L., Riksen N.P. (2023). The optimal healthy ranges of thyroid function defined by the risk of cardiovascular disease and mortality: Systematic review and individual participant data meta-analysis. Lancet Diabetes Endocrinol..

[30] Corona G., Croce L., Sparano C., Petrone L., Sforza A., Maggi M., Chiovato L., Rotondi M. (2021). Thyroid and heart, a clinically relevant relationship. J. Endocrinol. Investig..

[31] Yadalam A.K., Desai S.R., Razavi A.C., Gold M.E., Jain V., Vatsa N., Gold D., Ko Y.A., Haroun N., Nadkarni I. (2026). Safety and Tolerability of Short-Term Oral Triiodothyronine in Euthyroid Patients with Ischemic Heart Failure: A Phase I, Open-Label, Controlled Clinical Trial. Am. J. Cardiol..

[32] Borso M., Agretti P., Zucchi R., Saba A. (2022). Mass spectrometry in the diagnosis of thyroid disease and in the study of thyroid hormone metabolism. Mass Spectrom. Rev..

[33] Guo A., Song L.S. (2014). AutoTT: Automated detection and analysis of T-tubule architecture in cardiomyocytes. Biophys. J..

[34] Hong T., Yang H., Zhang S.S., Cho H.C., Kalashnikova M., Sun B., Zhang H., Bhargava A., Grabe M., Olgin J. (2014). Cardiac BIN1 folds T-tubule membrane, controlling ion flux and limiting arrhythmia. Nat. Med..

[35] Ibrahim M., Rao C., Athanasiou T., Yacoub M.H., Terracciano C.M. (2012). Mechanical unloading and cell therapy have a synergistic role in the recovery and regeneration of the failing heart. Eur. J. Cardiothorac. Surg..

[36] Wei S., Guo A., Chen B., Kutschke W., Xie Y.P., Zimmerman K., Weiss R.M., Anderson M.E., Cheng H., Song L.S. (2010). T-tubule remodeling during transition from hypertrophy to heart failure. Circ. Res..

[37] Carr A.N., Kranias E.G. (2002). Thyroid hormone regulation of calcium cycling proteins. Thyroid.

[38] Dulhunty A.F., Gage P.W., Lamb G.D. (1986). Differential effects of thyroid hormone on T-tubules and terminal cisternae in rat muscles: An electrophysiological and morphometric analysis. J. Muscle Res. Cell Motil..

[39] Yang X., Rodriguez M., Pabon L., Fischer K.A., Reinecke H., Regnier M., Sniadecki N.J., Ruohola-Baker H., Murry C.E. (2014). Tri-iodo-l-thyronine promotes the maturation of human cardiomyocytes-derived from induced pluripotent stem cells. J. Mol. Cell. Cardiol..

[40] Manfra O., Louey S., Jonker S.S., Perdreau-Dahl H., Frisk M., Giraud G.D., Thornburg K.L., Louch W.E. (2024). Augmenting workload drives T-tubule assembly in developing cardiomyocytes. J. Physiol..

[41] Hu J., Gao C., Wei C., Xue Y., Shao C., Hao Y., Gou L.T., Zhou Y., Zhang J., Ren S. (2019). RBFox2-miR-34a-Jph2 axis contributes to cardiac decompensation during heart failure. Proc. Natl. Acad. Sci. USA.

[42] Guo A., Wang Y., Chen B., Wang Y., Yuan J., Zhang L., Hall D., Wu J., Shi Y., Zhu Q. (2018). E-C coupling structural protein junctophilin-2 encodes a stress-adaptive transcription regulator. Science.

[43] Rajagopalan V., Ojamaa K., Gerdes A.M. (2025). Rethinking strategies for solving thyroid dysfunction at the heart of cardiovascular disease. Mol. Med..

[44] Kannan L., Shaw P.A., Morley M.P., Brandimarto J., Fang J.C., Sweitzer N.K., Cappola T.P., Cappola A.R. (2018). Thyroid Dysfunction in Heart Failure and Cardiovascular Outcomes. Circ. Heart Fail..

[45] Greco L.V., Charest A., Li Y., Udo-Bellner L., Ojamaa K., Gerdes A.M., Zhang Y. (2025). Failing hearts are more vulnerable to dobutamine and caffeine-induced ventricular arrhythmias: Ameliorated with dantrolene treatment. Heart Rhythm O2.

[46] Saba A., Donzelli R., Colligiani D., Raffaelli A., Nannipieri M., Kusmic C., Dos Remedios C.G., Simonides W.S., Iervasi G., Zucchi R. (2014). Quantification of thyroxine and 3,5,3′-triiodo-thyronine in human and animal hearts by a novel liquid chromatography-tandem mass spectrometry method. Horm. Metab. Res..

[47] Donzelli R., Colligiani D., Kusmic C., Sabatini M., Lorenzini L., Accorroni A., Nannipieri M., Saba A., Iervasi G., Zucchi R. (2016). Effect of Hypothyroidism and Hyperthyroidism on Tissue Thyroid Hormone Concentrations in Rat. Eur. Thyroid J..

[48] Ester M., Kriegel H.P., Sander J., Xu X. (1996). A density-based algorithm for discovering clusters in large spatial databases with noise. Proceedings of 2nd International Conference on Knowledge Discovery and Data Mining.

